# *catena*-Poly[[(4-formyl­benzoato-κ*O* ^1^)(isonicotinamide-κ*N* ^1^)zinc(II)]-μ-4-formyl­benzoato-κ^2^ *O* ^1^:*O* ^1′^]

**DOI:** 10.1107/S160053680904241X

**Published:** 2009-10-23

**Authors:** Tuncer Hökelek, Filiz Yılmaz, Barış Tercan, Mustafa Sertçelik, Hacali Necefoğlu

**Affiliations:** a Department of Physics, Hacettepe University, 06800 Beytepe, Ankara, Turkey; b Department of Chemistry, Faculty of Science, Anadolu University, 26470 Yenibağlar, Eskişehir, Turkey; c Department of Physics, Karabük University, 78050 Karabük, Turkey; d Department of Chemistry, Kafkas University, 63100 Kars, Turkey

## Abstract

In the title compound, [Zn(C_8_H_5_O_3_)_2_(C_6_H_6_N_2_O)]_*n*_, the Zn^II^ ion is tetrahedrally coordinated by two formyl­benzoate (FB) and one isonicotinamide (INA) ligands while symmetry-related FB ligands bridge adjacent Zn^II^ ions, forming polymeric chains along the *b* axis. The carboxyl­ate groups in the two FB ions are twisted away from the attached benzene ring by 9.07 (2) and 26.2 (2)°. The two benzene rings of the FB ions are oriented at a dihedral angle of 81.30 (5)°. In the crystal, adjacent polymeric chains inter­act *via* N—H⋯O and C—H⋯O hydrogen bonds, π–π contacts between the formyl­benzoate rings [centroid–centroid distance = 3.7736 (8) Å] and weak C—H⋯π inter­actions, forming a three-dimensional network.

## Related literature

For general background to niacin, see: Krishnamachari (1974 ▶). For the crystal structure of *N*,*N*-diethyl­nicotinamide, see: Bigoli *et al.* (1972 ▶). For related structures, see: Hökelek & Necefoğlu (1996 ▶); Hökelek *et al.* (2009 ▶). 
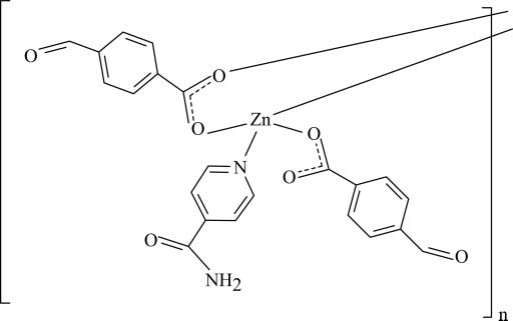


## Experimental

### 

#### Crystal data

[Zn(C_8_H_5_O_3_)_2_(C_6_H_6_N_2_O)]
*M* *_r_* = 485.74
Monoclinic, 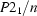

*a* = 13.3143 (2) Å
*b* = 6.7857 (1) Å
*c* = 21.3927 (3) Å
β = 91.458 (1)°
*V* = 1932.14 (5) Å^3^
*Z* = 4
Mo *K*α radiation
μ = 1.32 mm^−1^
*T* = 100 K
0.22 × 0.12 × 0.08 mm

#### Data collection

Bruker Kappa APEXII CCD area-detector diffractometer
Absorption correction: multi-scan (*SADABS*; Bruker, 2005 ▶) *T*_min_ = 0.829, *T*_max_ = 0.903
17841 measured reflections
4812 independent reflections
4086 reflections with *I* > 2σ(*I*)
*R*_int_ = 0.072

#### Refinement

*R*[*F* ^2^ > 2σ(*F* ^2^)] = 0.028
*wR*(*F* ^2^) = 0.080
*S* = 1.10
4812 reflections
297 parameters
H atoms treated by a mixture of independent and constrained refinement
Δρ_max_ = 0.47 e Å^−3^
Δρ_min_ = −0.39 e Å^−3^

### 

Data collection: *APEX2* (Bruker, 2007 ▶); cell refinement: *SAINT* (Bruker, 2007 ▶); data reduction: *SAINT*; program(s) used to solve structure: *SHELXS97* (Sheldrick, 2008 ▶); program(s) used to refine structure: *SHELXL97* (Sheldrick, 2008 ▶); molecular graphics: *ORTEP-3 for Windows* (Farrugia, 1997 ▶); software used to prepare material for publication: *WinGX* (Farrugia, 1999 ▶) and *PLATON* (Spek, 2009 ▶).

## Supplementary Material

Crystal structure: contains datablocks I, global. DOI: 10.1107/S160053680904241X/ci2939sup1.cif

Structure factors: contains datablocks I. DOI: 10.1107/S160053680904241X/ci2939Isup2.hkl

Additional supplementary materials:  crystallographic information; 3D view; checkCIF report

## Figures and Tables

**Table 1:** Selected bond lengths (Å)

Zn1—O1	1.9153 (11)
Zn1—O3	1.9723 (11)
Zn1—O4	1.9450 (10)
Zn1—N1	2.0270 (12)

**Table 2:** Hydrogen-bond geometry (Å, °)

*D*—H⋯*A*	*D*—H	H⋯*A*	*D*⋯*A*	*D*—H⋯*A*
N2—H2*A*⋯O2^i^	0.86	2.08	2.9242 (17)	165
N2—H2*B*⋯O2^ii^	0.86	2.11	2.9439 (17)	163
C4—H4⋯O5^iii^	0.93	2.41	3.298 (2)	160
C6—H6⋯O7^iv^	0.93	2.50	3.223 (2)	135
C15—H15⋯O6^iv^	0.93	2.32	3.2049 (19)	159
C16—H16⋯O2^ii^	0.93	2.44	3.3541 (18)	169
C3—H3⋯*Cg*1	0.93	2.73	3.6332 (17)	163

Symmetry codes: (i) 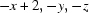; (ii) 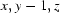; (iii) 
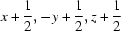
; (iv) 
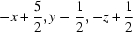
. *Cg*1 is the centroid of the C9–C14 ring.

**Fig. 1.**
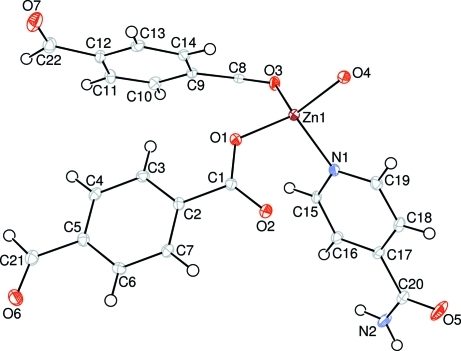
The asymmetric unit of the title compound with the atom-numbering scheme. Displacement ellipsoids are drawn at the 50% probability level.

**Fig. 2.**
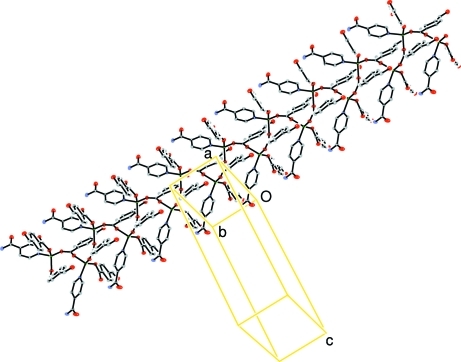
Part of a polymeric chain of the title compound.

## supplementary crystallographic information

### Comment

As a part of our ongoing investigation on transition metal complexes of nicotinamide (NA), one form of niacin (Krishnamachari, 1974), and/or the nicotinic acid derivative *N*,*N*-diethylnicotinamide (DENA), an important respiratory stimulant (Bigoli *et al.*, 1972), the title compound was synthesized and its crystal structure is reported herein.

In the crystal structure of the title compound, each Zn^II^ ion is coordinated by two formylbenzoate (FB) and one isonicotinamide (INA) ligands (Fig. 1), while symmetry related FB ligands bridge the Zn^II^ ions forming polymeric chains along the *b* axis (Fig.2). The structures of similar complexes of Zn^II^ ion, [Zn_2_(C_10_H_14_N_2_O)_2_(C_7_H_5_O_3_)_4_].2H_2_O (Hökelek & Necefouglu, 1996) and [Zn(C_9_H_10_NO_2_)_2_(C_6_H_6_N_2_O)(H_2_O)_2_] (Hökelek *et al.*, 2009) have also been reported.

The average Zn—O bond length (Table 1) is 1.9442 (11) Å and the Zn1 atom is displaced out of the least-squares planes of the carboxylate groups (O1/C1/O2) and (O3/C8/O4*) by 0.687 (5) Å and 0.703 (2) Å, respectively. The O1/C1/O2 and O3/C8/O4* carboxylate planes form dihedral angles of 9.07 (2)° and 26.2 (2)°, respectively, with benzene rings A(C2-C7) and B(C9-C14), while the angles between rings A, B and C (N1/C15-C19) are A/B = 81.30 (5), A/C = 63.17 (5) and B/C = 46.11 (5)°.

In the crystal structure, N—H···O and C—H···O hydrogen bonds (Table 2) link adjacent chains into a three-dimensional network. In addition, π–π contacts between symmetry related A(C2-C7) formylbenzoate rings at (x, y, z) and (5/2-x, -1/2+y, 1/2-z)/(5/2-x, 1/2+y, 1/2-z) with a centroid-to-centroid distance of 3.7736 (8) Å, and weak C—H···π interaction (Table 2) involving the B(C9-C14) ring stabilize the structure.

### Experimental

The title compound was prepared by the reaction of ZnSO_4_.H_2_O (0.90 g, 5 mmol) in H_2_O (25 ml) and INA (1.22 g, 10 mmol) in H_2_O (40 ml) with sodium 4-formylbenzoate (1.72 g, 10 mmol) in H_2_O (50 ml). The mixture was filtered and set aside to crystallize at ambient temperature for several days, giving colourless single crystals.

### Refinement

Atoms H21 and H22 (for methine) were located in a difference Fourier map and refined isotropically. The remaining H atoms were positioned geometrically with N-H = 0.86 Å (for NH_2_) and C-H = 0.93 Å for aromatic H atoms and constrained to ride on their parent atoms, with *U*_iso_(H) = 1.2*U*_eq_(C,N).

### Crystal data

**Table:**

[Zn(C_8_H_5_O_3_)_2_(C_6_H_6_N_2_O)]	*F*(000) = 992
*M**_r_* = 485.74	*D*_x_ = 1.670 Mg m^−^^3^
Monoclinic, *P*2_1_/*n*	Mo *K*α radiation, λ = 0.71073 Å
Hall symbol: -P 2yn	Cell parameters from 9448 reflections
*a* = 13.3143 (2) Å	θ = 3.1–28.4°
*b* = 6.7857 (1) Å	µ = 1.32 mm^−^^1^
*c* = 21.3927 (3) Å	*T* = 100 K
β = 91.458 (1)°	Needle, colourless
*V* = 1932.14 (5) Å^3^	0.22 × 0.12 × 0.08 mm
*Z* = 4	

### Data collection

**Table:**

Bruker Kappa APEXII CCD area-detector diffractometer	4812 independent reflections
Radiation source: fine-focus sealed tube	4086 reflections with *I* > 2σ(*I*)
graphite	*R*_int_ = 0.072
φ and ω scans	θ_max_ = 28.4°, θ_min_ = 1.8°
Absorption correction: multi-scan (*SADABS*; Bruker, 2005)	*h* = −17→14
*T*_min_ = 0.829, *T*_max_ = 0.903	*k* = −8→9
17841 measured reflections	*l* = −28→28

### Refinement

**Table:**

Refinement on *F*^2^	Primary atom site location: structure-invariant direct methods
Least-squares matrix: full	Secondary atom site location: difference Fourier map
*R*[*F*^2^ > 2σ(*F*^2^)] = 0.028	Hydrogen site location: inferred from neighbouring sites
*wR*(*F*^2^) = 0.080	H atoms treated by a mixture of independent and constrained refinement
*S* = 1.10	*w* = 1/[σ^2^(*F*_o_^2^) + (0.0394*P*)^2^ + 0.0092*P*] where *P* = (*F*_o_^2^ + 2*F*_c_^2^)/3
4812 reflections	(Δ/σ)_max_ = 0.001
297 parameters	Δρ_max_ = 0.47 e Å^−^^3^
0 restraints	Δρ_min_ = −0.39 e Å^−^^3^

### Special details

**Table:**

Geometry. All e.s.d.'s (except the e.s.d. in the dihedral angle between two l.s. planes) are estimated using the full covariance matrix. The cell e.s.d.'s are taken into account individually in the estimation of e.s.d.'s in distances, angles and torsion angles; correlations between e.s.d.'s in cell parameters are only used when they are defined by crystal symmetry. An approximate (isotropic) treatment of cell e.s.d.'s is used for estimating e.s.d.'s involving l.s. planes.
Refinement. Refinement of *F*^2^ against ALL reflections. The weighted *R*-factor *wR* and goodness of fit *S* are based on *F*^2^, conventional *R*-factors *R* are based on *F*, with *F* set to zero for negative *F*^2^. The threshold expression of *F*^2^ > σ(*F*^2^) is used only for calculating *R*-factors(gt) *etc*. and is not relevant to the choice of reflections for refinement. *R*-factors based on *F*^2^ are statistically about twice as large as those based on *F*, and *R*- factors based on ALL data will be even larger.

### Fractional atomic coordinates and isotropic or equivalent isotropic displacement parameters (Å^2^)

**Table:**

	*x*	*y*	*z*	*U*_iso_*/*U*_eq_	
Zn1	0.816160 (13)	0.51300 (2)	0.187880 (8)	0.01058 (7)	
O1	0.95052 (8)	0.60767 (16)	0.20123 (5)	0.0149 (2)	
O2	1.00643 (8)	0.55706 (16)	0.10502 (5)	0.0149 (2)	
O3	0.77863 (8)	0.37932 (15)	0.26575 (5)	0.0134 (2)	
O4	0.70741 (8)	0.67550 (14)	0.15404 (5)	0.0137 (2)	
O5	0.83582 (11)	−0.07543 (19)	−0.07713 (6)	0.0312 (3)	
O6	1.49773 (9)	0.61367 (17)	0.23858 (5)	0.0199 (3)	
O7	1.10801 (11)	0.97953 (16)	0.45862 (6)	0.0236 (3)	
N1	0.81632 (9)	0.29546 (17)	0.12295 (6)	0.0112 (3)	
N2	0.91501 (11)	−0.28715 (18)	−0.01158 (6)	0.0182 (3)	
H2A	0.9269	−0.3695	−0.0410	0.022*	
H2B	0.9348	−0.3125	0.0262	0.022*	
C1	1.02044 (11)	0.5821 (2)	0.16202 (7)	0.0110 (3)	
C2	1.12564 (11)	0.5862 (2)	0.18971 (7)	0.0109 (3)	
C3	1.14051 (12)	0.5879 (2)	0.25423 (7)	0.0126 (3)	
H3	1.0857	0.5867	0.2803	0.015*	
C4	1.23802 (12)	0.5913 (2)	0.27978 (7)	0.0131 (3)	
H4	1.2487	0.5906	0.3229	0.016*	
C5	1.31866 (11)	0.5957 (2)	0.24033 (7)	0.0122 (3)	
C6	1.30379 (12)	0.5942 (2)	0.17551 (7)	0.0143 (3)	
H6	1.3585	0.5979	0.1494	0.017*	
C7	1.20746 (12)	0.5872 (2)	0.15043 (7)	0.0134 (3)	
H7	1.1971	0.5832	0.1073	0.016*	
C8	0.81930 (11)	0.3293 (2)	0.31722 (7)	0.0115 (3)	
C9	0.89783 (12)	0.4527 (2)	0.34954 (7)	0.0114 (3)	
C10	0.96360 (12)	0.3649 (2)	0.39317 (7)	0.0149 (3)	
H10	0.9603	0.2299	0.4004	0.018*	
C11	1.03354 (13)	0.4785 (2)	0.42548 (8)	0.0162 (3)	
H11	1.0792	0.4188	0.4531	0.019*	
C12	1.03609 (12)	0.6823 (2)	0.41700 (7)	0.0140 (3)	
C13	0.97104 (12)	0.7698 (2)	0.37288 (7)	0.0138 (3)	
H13	0.9730	0.9053	0.3666	0.017*	
C14	0.90409 (12)	0.6556 (2)	0.33872 (7)	0.0122 (3)	
H14	0.8627	0.7138	0.3083	0.015*	
C15	0.86757 (11)	0.1289 (2)	0.13520 (7)	0.0125 (3)	
H15	0.8924	0.1074	0.1757	0.015*	
C16	0.88489 (13)	−0.0119 (2)	0.09003 (8)	0.0139 (3)	
H16	0.9213	−0.1251	0.1000	0.017*	
C17	0.84719 (12)	0.0179 (2)	0.02948 (8)	0.0126 (3)	
C18	0.79165 (12)	0.1872 (2)	0.01756 (7)	0.0147 (3)	
H18	0.7639	0.2097	−0.0221	0.018*	
C19	0.77770 (12)	0.3220 (2)	0.06473 (7)	0.0136 (3)	
H19	0.7404	0.4350	0.0561	0.016*	
C20	0.86597 (12)	−0.1204 (2)	−0.02441 (7)	0.0157 (3)	
C21	1.42128 (13)	0.6036 (2)	0.26818 (8)	0.0161 (3)	
H21	1.4266 (13)	0.600 (3)	0.3157 (9)	0.028 (5)*	
C22	1.10601 (13)	0.8010 (2)	0.45686 (8)	0.0188 (4)	
H22	1.1561 (13)	0.714 (3)	0.4842 (9)	0.020 (5)*	

### Atomic displacement parameters (Å^2^)

**Table:**

	*U*^11^	*U*^22^	*U*^33^	*U*^12^	*U*^13^	*U*^23^
Zn1	0.01001 (11)	0.01290 (10)	0.00882 (11)	0.00080 (6)	−0.00023 (7)	−0.00111 (6)
O1	0.0092 (6)	0.0225 (6)	0.0129 (5)	−0.0007 (4)	0.0003 (4)	−0.0045 (4)
O2	0.0144 (6)	0.0195 (5)	0.0106 (5)	0.0021 (5)	−0.0019 (5)	−0.0020 (4)
O3	0.0120 (6)	0.0184 (5)	0.0098 (5)	−0.0013 (4)	−0.0021 (4)	0.0016 (4)
O4	0.0134 (6)	0.0141 (5)	0.0135 (5)	0.0030 (4)	−0.0013 (5)	−0.0022 (4)
O5	0.0524 (9)	0.0310 (7)	0.0097 (6)	0.0163 (7)	−0.0092 (6)	−0.0050 (5)
O6	0.0130 (6)	0.0295 (6)	0.0172 (6)	−0.0013 (5)	−0.0005 (5)	0.0005 (5)
O7	0.0279 (8)	0.0248 (6)	0.0181 (7)	−0.0107 (5)	0.0007 (6)	−0.0038 (5)
N1	0.0094 (7)	0.0145 (6)	0.0096 (6)	−0.0011 (5)	−0.0002 (5)	0.0003 (5)
N2	0.0256 (8)	0.0193 (6)	0.0094 (6)	0.0055 (6)	−0.0018 (6)	−0.0037 (5)
C1	0.0111 (8)	0.0103 (6)	0.0115 (7)	0.0008 (6)	−0.0008 (6)	−0.0005 (6)
C2	0.0111 (8)	0.0102 (6)	0.0113 (7)	0.0000 (6)	−0.0016 (6)	0.0001 (6)
C3	0.0128 (8)	0.0145 (7)	0.0106 (7)	0.0009 (6)	0.0017 (6)	0.0001 (6)
C4	0.0164 (8)	0.0143 (7)	0.0085 (7)	0.0000 (6)	−0.0008 (6)	−0.0002 (6)
C5	0.0121 (8)	0.0110 (7)	0.0135 (8)	0.0003 (6)	−0.0016 (6)	0.0002 (6)
C6	0.0113 (8)	0.0190 (7)	0.0127 (7)	−0.0008 (6)	0.0028 (6)	−0.0003 (6)
C7	0.0143 (8)	0.0183 (7)	0.0076 (7)	0.0006 (6)	−0.0008 (6)	0.0006 (6)
C8	0.0096 (8)	0.0138 (7)	0.0113 (7)	0.0029 (6)	0.0015 (6)	−0.0023 (6)
C9	0.0098 (8)	0.0154 (7)	0.0091 (7)	−0.0002 (6)	0.0015 (6)	−0.0020 (6)
C10	0.0173 (9)	0.0137 (7)	0.0137 (8)	0.0002 (6)	−0.0001 (7)	−0.0003 (6)
C11	0.0144 (9)	0.0206 (8)	0.0134 (8)	0.0021 (6)	−0.0043 (7)	−0.0003 (6)
C12	0.0113 (8)	0.0191 (7)	0.0117 (7)	−0.0021 (6)	−0.0004 (6)	−0.0029 (6)
C13	0.0149 (8)	0.0136 (7)	0.0129 (7)	−0.0015 (6)	0.0041 (6)	−0.0024 (6)
C14	0.0111 (8)	0.0164 (7)	0.0090 (7)	0.0020 (6)	0.0012 (6)	0.0004 (6)
C15	0.0116 (8)	0.0171 (7)	0.0087 (7)	0.0004 (6)	−0.0023 (6)	0.0008 (6)
C16	0.0157 (9)	0.0140 (7)	0.0120 (8)	0.0026 (6)	−0.0017 (7)	−0.0003 (5)
C17	0.0147 (8)	0.0143 (7)	0.0088 (8)	−0.0022 (6)	0.0006 (6)	−0.0003 (5)
C18	0.0169 (9)	0.0176 (7)	0.0094 (7)	−0.0006 (6)	−0.0029 (6)	0.0014 (6)
C19	0.0130 (8)	0.0150 (7)	0.0128 (7)	0.0019 (6)	−0.0011 (6)	0.0029 (6)
C20	0.0180 (9)	0.0185 (7)	0.0106 (7)	0.0001 (6)	−0.0013 (7)	−0.0024 (6)
C21	0.0173 (9)	0.0171 (7)	0.0139 (8)	0.0003 (6)	−0.0024 (7)	−0.0003 (6)
C22	0.0174 (9)	0.0260 (8)	0.0131 (8)	−0.0046 (7)	0.0007 (7)	−0.0018 (7)

### Geometric parameters (Å, °)

**Table:**

Zn1—O1	1.9153 (11)	C7—C6	1.378 (2)
Zn1—O3	1.9723 (11)	C7—H7	0.93
Zn1—O4	1.9450 (10)	C8—O4^ii^	1.2669 (18)
Zn1—N1	2.0270 (12)	C8—C9	1.495 (2)
O1—C1	1.2807 (18)	C9—C10	1.397 (2)
O2—C1	1.2405 (18)	C9—C14	1.3990 (19)
O3—C8	1.2611 (17)	C10—H10	0.93
O4—C8^i^	1.2669 (18)	C11—C10	1.381 (2)
O5—C20	1.2261 (18)	C11—H11	0.93
O6—C21	1.214 (2)	C12—C11	1.395 (2)
O7—C22	1.2124 (19)	C12—C13	1.397 (2)
N1—C15	1.3427 (18)	C12—C22	1.484 (2)
N1—C19	1.3474 (18)	C13—C14	1.377 (2)
N2—C20	1.332 (2)	C13—H13	0.93
N2—H2A	0.86	C14—H14	0.93
N2—H2B	0.86	C15—H15	0.93
C2—C1	1.507 (2)	C16—C15	1.382 (2)
C2—C3	1.390 (2)	C16—C17	1.392 (2)
C2—C7	1.393 (2)	C16—H16	0.93
C3—C4	1.396 (2)	C18—C17	1.386 (2)
C3—H3	0.93	C18—H18	0.93
C4—H4	0.93	C19—C18	1.378 (2)
C5—C4	1.383 (2)	C19—H19	0.93
C5—C6	1.396 (2)	C20—C17	1.512 (2)
C5—C21	1.478 (2)	C21—H21	1.018 (19)
C6—H6	0.93	C22—H22	1.057 (17)
			
O1—Zn1—O3	106.50 (4)	C14—C9—C8	121.23 (13)
O1—Zn1—O4	123.30 (5)	C9—C10—H10	120.0
O1—Zn1—N1	109.22 (5)	C11—C10—C9	119.95 (14)
O3—Zn1—N1	104.39 (5)	C11—C10—H10	120.0
O4—Zn1—O3	111.89 (5)	C10—C11—C12	120.45 (15)
O4—Zn1—N1	99.88 (5)	C10—C11—H11	119.8
C1—O1—Zn1	123.27 (10)	C12—C11—H11	119.8
C8—O3—Zn1	138.48 (10)	C11—C12—C13	119.50 (14)
C8^i^—O4—Zn1	120.13 (10)	C11—C12—C22	118.70 (15)
C15—N1—Zn1	119.26 (10)	C13—C12—C22	121.77 (14)
C15—N1—C19	118.22 (13)	C12—C13—H13	120.0
C19—N1—Zn1	121.95 (10)	C14—C13—C12	120.09 (14)
C20—N2—H2A	120.0	C14—C13—H13	120.0
C20—N2—H2B	120.0	C9—C14—H14	119.8
H2A—N2—H2B	120.0	C13—C14—C9	120.42 (14)
O1—C1—C2	115.07 (13)	C13—C14—H14	119.8
O2—C1—O1	124.67 (14)	N1—C15—C16	122.57 (14)
O2—C1—C2	120.25 (14)	N1—C15—H15	118.7
C3—C2—C1	119.87 (14)	C16—C15—H15	118.7
C3—C2—C7	120.36 (14)	C15—C16—C17	119.19 (14)
C7—C2—C1	119.76 (13)	C15—C16—H16	120.4
C2—C3—C4	119.77 (14)	C17—C16—H16	120.4
C2—C3—H3	120.1	C16—C17—C20	123.82 (13)
C4—C3—H3	120.1	C18—C17—C16	118.00 (14)
C3—C4—H4	120.3	C18—C17—C20	118.16 (14)
C5—C4—C3	119.36 (14)	C17—C18—H18	120.1
C5—C4—H4	120.3	C19—C18—C17	119.77 (14)
C4—C5—C6	120.90 (14)	C19—C18—H18	120.1
C4—C5—C21	118.62 (14)	N1—C19—C18	122.20 (14)
C6—C5—C21	120.48 (14)	N1—C19—H19	118.9
C5—C6—H6	120.2	C18—C19—H19	118.9
C7—C6—C5	119.60 (14)	O5—C20—N2	123.26 (15)
C7—C6—H6	120.2	O5—C20—C17	119.35 (14)
C2—C7—H7	120.0	N2—C20—C17	117.39 (13)
C6—C7—C2	119.98 (14)	O6—C21—C5	124.78 (15)
C6—C7—H7	120.0	O6—C21—H21	119.0 (11)
O3—C8—O4^ii^	121.72 (14)	C5—C21—H21	116.3 (11)
O3—C8—C9	122.19 (13)	O7—C22—C12	124.98 (17)
O4^ii^—C8—C9	116.01 (13)	O7—C22—H22	121.9 (9)
C10—C9—C8	119.27 (13)	C12—C22—H22	113.1 (9)
C10—C9—C14	119.45 (14)		
			
O3—Zn1—O1—C1	132.27 (11)	C21—C5—C4—C3	178.69 (13)
O4—Zn1—O1—C1	−96.44 (12)	C4—C5—C6—C7	−0.3 (2)
N1—Zn1—O1—C1	20.07 (13)	C21—C5—C6—C7	−179.85 (14)
O1—Zn1—O3—C8	−6.56 (15)	C4—C5—C21—O6	−177.81 (15)
O4—Zn1—O3—C8	−143.97 (14)	C6—C5—C21—O6	1.7 (2)
N1—Zn1—O3—C8	108.94 (15)	C2—C7—C6—C5	1.5 (2)
O1—Zn1—O4—C8^i^	−60.90 (13)	O3—C8—C9—C10	−158.23 (15)
O3—Zn1—O4—C8^i^	68.17 (12)	O3—C8—C9—C14	24.3 (2)
N1—Zn1—O4—C8^i^	178.16 (11)	O4^ii^—C8—C9—C10	24.8 (2)
O1—Zn1—N1—C15	62.86 (12)	O4^ii^—C8—C9—C14	−152.61 (15)
O1—Zn1—N1—C19	−108.32 (12)	C8—C9—C10—C11	−177.06 (15)
O3—Zn1—N1—C15	−50.72 (12)	C14—C9—C10—C11	0.4 (2)
O3—Zn1—N1—C19	138.10 (12)	C8—C9—C14—C13	174.28 (14)
O4—Zn1—N1—C15	−166.53 (11)	C10—C9—C14—C13	−3.2 (2)
O4—Zn1—N1—C19	22.29 (13)	C12—C11—C10—C9	2.9 (3)
Zn1—O1—C1—O2	25.4 (2)	C13—C12—C11—C10	−3.6 (3)
Zn1—O1—C1—C2	−155.21 (9)	C22—C12—C11—C10	174.41 (16)
Zn1—O3—C8—O4^ii^	−147.48 (12)	C11—C12—C13—C14	0.9 (2)
Zn1—O3—C8—C9	35.8 (2)	C22—C12—C13—C14	−177.08 (15)
Zn1—N1—C15—C16	−169.24 (12)	C11—C12—C22—O7	−171.97 (17)
C19—N1—C15—C16	2.3 (2)	C13—C12—C22—O7	6.0 (3)
Zn1—N1—C19—C18	169.38 (12)	C12—C13—C14—C9	2.5 (2)
C15—N1—C19—C18	−1.9 (2)	C17—C16—C15—N1	−0.7 (2)
C3—C2—C1—O1	9.2 (2)	C15—C16—C17—C18	−1.4 (2)
C3—C2—C1—O2	−171.42 (14)	C15—C16—C17—C20	176.70 (15)
C7—C2—C1—O1	−171.18 (13)	C19—C18—C17—C16	1.7 (2)
C7—C2—C1—O2	8.2 (2)	C19—C18—C17—C20	−176.45 (14)
C1—C2—C3—C4	179.87 (13)	N1—C19—C18—C17	−0.1 (2)
C7—C2—C3—C4	0.2 (2)	N2—C20—C17—C16	5.3 (2)
C1—C2—C7—C6	178.94 (14)	N2—C20—C17—C18	−176.65 (15)
C3—C2—C7—C6	−1.4 (2)	O5—C20—C17—C16	−174.85 (17)
C2—C3—C4—C5	0.9 (2)	O5—C20—C17—C18	3.2 (2)
C6—C5—C4—C3	−0.8 (2)		

Symmetry codes: (i) −*x*+3/2, *y*+1/2, −*z*+1/2; (ii) −*x*+3/2, *y*−1/2, −*z*+1/2.

### Hydrogen-bond geometry (Å, °)

**Table:**

*D*—H···*A*	*D*—H	H···*A*	*D*···*A*	*D*—H···*A*
N2—H2A···O2^iii^	0.86	2.08	2.9242 (17)	165
N2—H2B···O2^iv^	0.86	2.11	2.9439 (17)	163
C4—H4···O5^v^	0.93	2.41	3.298 (2)	160
C6—H6···O7^vi^	0.93	2.50	3.223 (2)	135
C15—H15···O6^vi^	0.93	2.32	3.2049 (19)	159
C16—H16···O2^iv^	0.93	2.44	3.3541 (18)	169
C3—H3···Cg1	0.93	2.73	3.6332 (17)	163

Symmetry codes: (iii) −*x*+2, −*y*, −*z*; (iv) *x*, *y*−1, *z*; (v) *x*+1/2, −*y*+1/2, *z*+1/2; (vi) −*x*+5/2, *y*−1/2, −*z*+1/2.
